# Clinical utility of microRNA-451 as diagnostic biomarker for human cancers

**DOI:** 10.1042/BSR20180653

**Published:** 2019-01-15

**Authors:** Zhanzhan Li, Yanyan Li, Jun Fu, Na Li, Liangfang Shen

**Affiliations:** 1 Department of Oncology, Xiangya Hospital, Central South University, Changsha, Hunan Province 410008, China; 2 Department of Outpatient, Xiangya Hospital, Central South University, Changsha, Hunan Province 410008, China

**Keywords:** Cancer, MicroRNA-451, Meta-analysis, Tumor marker

## Abstract

We conducted comprehensive analyses to assess the diagnostic ability of miRNA-451 in cancers. A systematic online search was conducted in PubMed, Web of Science, China’s national knowledge infrastructure, and VIP databases from inception to July 31, 2017. The bivariate random effect model was used for calculating sensitivity, specificity, positive likelihood ratio, negative likelihood ratio, diagnostic odds ratio, and area under cure (AUC). The whole pooled sensitivity and specificity were 0.85 (0.77–0.90) and 0.85 (0.78–0.90) with their 95% confidence interval (95%CI), respectively. The pooled AUC was 0.91 (95%CI: 0.89–0.94). Positive likelihood ratio was 5.57 (95%CI: 3.74–8.31), negative likelihood ratio was 0.18 (95%CI: 0.11–0.28), and diagnostic odds ratio was 31.33 (95%CI: 15.19–64.61). Among Asian population, the sensitivity and specificity were 0.85 (95%CI: 0.77–0.91) and 0.86 (95%CI: 0.78–0.91), respectively. The positive likelihood ratio and negative likelihood ratio were 5.87 (95%CI: 3.78–9.12) and 0.17 (95%CI: 0.11–0.28). The diagnostic odds ratio and AUC were 34.31 (15.51–75.91) and 0.92 (0.89–0.94). The pooled sensitivity, specificity, positive likelihood ratio, negative likelihood ratio, diagnostic odds ratio, and AUC for digestive system cancer were 0.83, 0.88, 6.87, 0.20, 35.13, and 0.92, respectively. The other cancers were 0.87, 0.81, 4.55, 0.16, 28.51, and 0.90, respectively. For sample source, the results still remain consistent. Our results indicated miRNA-451 has a moderate diagnostic ability for cancers, and could be a potential early screening biomarker, and considered as an adjuvant diagnostic index when being combined with other clinical examinations.

## Introduction

Cancer is a major public health issue all over the world, and incidence rates have increased in developing and developed countries. It was estimated that cancers had caused more than 8,000,000 deaths worldwide only in 2013 and became the second leading death cause in this year [1]. The incidence of cancer is higher in developing countries. However, higher mortality is observed in developing countries. Although great work has been made for cancer prevention and treatment, disease burden of cancer is still increasing because of the trend of global aging [2]. It was reported that the 5-year overall survival rate of patients with cancer was approximately 50% [3]. In fact, the prevention and treatment of cancer largely depend on early screening and detection. It is extremely difficult to achieve curable treatment effects for patients with end-stage cancer. Therefore, it is of great importance for patients to conduct early screening, and find early tumor biomarkers appear so significant.

The microRNA (miRNA) is a family of mature non-coding small RNAs. As a member of miRNA family, binding to the 3’-untranslated regions (3’UTR) of the target mRNA induces translational repression or miRNA degradation of many genes. Many studies had reported miRNA can regulate many cancer cell proliferation, growth, and development via different signaling pathways [4]. The miRNAs are highly stable and abundant in plasma, serum, and other body fluids. Moreover, miRNA signatures in blood are similar in men and women, as well as individuals of different ages and other factors. The miR-451 is one of the most conservative miRNAs and has important clinical application value. Significant expression difference of miRAN-451 was observed in a variety of cancers such as esophageal cancer [5], gastric cancer [6], renal cell carcinoma [7], hepatocellular carcinoma [8], colorectal cancer [9], and breast cancer [10]. These results indicate miRNA-451 is an important regulating factor in the occurrence and development of cancer and could be a potential tumor biomarker of early-stage screening. Currently, many studies have reported that the miRNA-451 expression was different in different cancers. The accuracy diagnostic ability still remained unclear because single study has some limitations such as sample size, cancer type, and population. We systematically conducted online searches and collected data from different cancers, with the aim of giving a comprehensive assessment for clinical diagnostic value of miRNA-451.

## Methods

### Search strategy

We performed a systematic search in PubMed, Web of Science, China’s national knowledge infrastructure, and VIP databases from inception to April 15, 2018. Both Medical Subject Headings (MeSHs) and key words were used to obtain potential studies. The following search words were adopted in combinations: (‘microRNA-451’ OR ‘miRNA-451’ OR ‘miR-451’ OR ‘has-mir-451’) AND (‘cancer’ OR ‘tumor’ OR ‘neoplasms’ OR ‘carcinoma’). We also retrieved the lists of articles and reviews for potentially eligible literatures.

### Criterion for inclusion and exclusion

Inclusion criteria: (1) study must be conducted among human, and type of tumor was confirmed by the pathology standard; (2) studies focused on the microRNA-451 in patients with tumor, and evaluated the diagnostic ability of microRNA-451; (3) study provided sufficient data to allow the calculation of diagnostic index, including true positive (TP), false positive (FP), false negative (FN), and true negative (TN). (4) Studies performed in *vitro, vivo*, and animals were excluded; the latest publication was used for duplicates; comments, letter, cases, and reviews were excluded as well as those without effective information.

### Data extraction

A standardized excel sheet was used for data extraction. Two investigators independently conducted data extraction, and disputes were solved by the third author (S.L.F.). For each included study, the following information was extracted: the first author, year of publication, region, type of cancer, source of sample, methods of examination, gold standard method, sample size (case/control), sensitivity, specificity, and all four fold values (TP, FP, FN, and TN).

### Assessment of quality

We used the updated quality assessment of diagnostic accuracy studies 2 to assess the quality of included studies [11]. This scale tool includes five key items: patient’s selection, index test, reference standard, and flow patients and timing of the index tests and reference standard. Each item consists of two subitems: risk of bias and applicability. The signaling questions are answered as ‘yes’, ‘no’, or ‘unclear’ and are phrased such that ‘no’ means high risk of bias, ‘yes’ indicates low risk of bias, and ‘unclear’ indicates unclear risk of bias.

### Statistical analysis

We firstly examined whether there was threshold value effect by Spearman correlation or not. If threshold value effect exists, we will conduct our analyses through hierarchical summary receive operating characteristic [12,13]. The bivariate mixed effects were used without threshold value effect [14]. Heterogeneity within studies was evaluated by the Chi-square test and *I*^2^ statistics, *I*^2^ > 50% or *P*<0.05 indicated significant heterogeneity [15]. We used random effects to combine the following parameters: sensitivity, specificity, positive likelihood ratios (PLRs) and negative likelihood ratios (NLRs) (PLR > 10, NLR < 0.1: exclusion and confirmation; PLR > 10, NLR > 0.1: confirmation only; PLR < 10, NLR < 0.1 exclusion only; PLR < 10, NLR > 0.1: no exclusion or confirmation), and diagnostic odds ratios (DORs) with 95% confidence intervals (CIs). We also conducted subgroup analysis in different settings: population, cancer types (digestive system vs others), and sample sources (serum or plasma). We also calculated the area under the summary receiver operator characteristic cure (SROC) and Fagan plots to evaluate the diagnostic ability of miR-451. The area under cure (AUC) of 1.0 means perfect diagnostic ability, while an AUC close to 0.5 indicates a poor diagnostic ability [16]. We used linear regression test for funnel plot asymmetry [17]. All analyses were conducted on Stata 14.0 software (StataCorp LP, College Station, TX, U.S.A.). *P*<0.05 indicated statistical significance.

## Results

### Study selection

**Figure 1:**
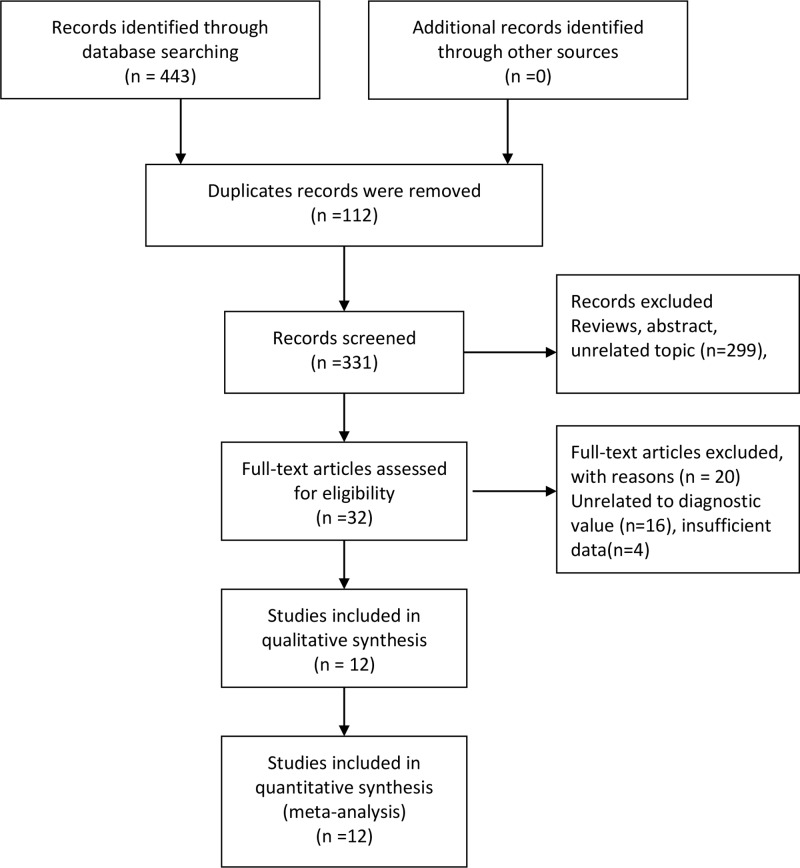
Flow diagram of studies selection

As presented in the flow Figure 1, our initial search returned 443 records, and we did not obtain additional records through other sources. After removing 112 duplicates records, we got 331 records for further screening. A total of 229 records were excluded because of some reviews and unrelated topic records. We prepared 32 full-text articles for potential eligibility. A total of 16 studies with unrelated to diagnostic value and 4 records with insufficient data were excluded. Finally, 10 articles with 12 studies data entered into qualitative and quantitative synthesis [5–10,18–21].

### General characteristics of included studies

Tables 1 and 2 presented the general characteristics of included studies. These studies were published from 2012 to 2017. Nine of ten studies were from Asian population, and only one study was conducted among European. Of ten studies, three studies were for esophageal cancer, three for gastric cancer, two for breast cancer, one for thyroid carcinoma, one for renal cell carcinoma, and one for hepatocellular carcinoma. The tissue samples were mainly from serum and plasma. But two samples were from saliva and one from feces. Two studies detected miRNA expression via Real-time PCR, and the rest used qRT-PCR. All cancers were confirmed through tissue pathology. The sample size ranged from 45 to 150, with total number of 1177. The sensitivity of included studies was from 51 to 96%, and the specificity was from 58 to 97%. The Tables 1 and 2 presented the specific details.

**Table 1:** General characteristics of included studies in the meta-analysis

Author	Year of publication	Region	Type of cancer	Source of sample	Methods of examination	Gold standard
Zhang	2015	Asia	Thyroid carcinoma	Serum	qRT-PCR	Tissue pathology
Yang	2017	Asia	Esophageal cancer	Serum	qRT-PCR	Tissue pathology
Xie	2013	Asia	Esophageal cancer	Saliva	qRT-PCR	Tissue pathology
Xie	2013	Asia	Esophageal cancer	Saliva	qRT-PCR	Tissue pathology
Zhu	2014	Asia	Gastric cancer	Plasma	qRT-PCR	Tissue pathology
Zhu	2014	Asia	Gastric cancer	Plasma	qRT-PCR	Tissue pathology
Redova	2012	Europe	Renal cell carcinoma	Serum	qRT-PCR	Tissue pathology
Pei	2014	Asia	Hepatocellular carcinoma	Serum	Real-time PCR	Tissue pathology
Konishi	2012	Asia	Gastric cancer	Plasma	qRT-PCR	Tissue pathology
Phua	2014	Asia	Colorectal cancer	Feces	Real-time PCR	Tissue pathology
Ng	2013	Asia	Breast cancer	Plasma	qRT-PCR	Tissue pathology
Luo	2014	Asia	Breast cancer	Serum	qRT-PCR	Tissue pathology

**Table 2:** Parameters of included studies in the meta-analysis

Author	Year	Sample size (case/control)	Total	TP	FP	FN	TN	Sensitivity (%)	Specificity (%)
Zhang	2015	60/60	120	47	18	13	42	78	70
Yang	2017	50/20	70	44	3	6	17	88	85
Xie	2013	39/19	58	20	3	19	16	85	58
Xie	2013	39/19	58	33	8	6	11	51	84
Zhu	2014	48/102	150	33	11	15	91	81	83
Zhu	2014	72/18	90	58	3	14	15	69	89
Redova	2012	90/35	125	73	8	17	27	81	77
Pei	2014	66/40	106	63	7	3	33	95	82
Konishi	2012	56/30	86	54	0	2	30	96	97
Phua	2014	17/28	45	15	0	2	28	88	97
Ng	2013	70/50	120	58	4	12	46	83	92
Luo	2014	60/29	89	56	6	4	23	93	79

### Assessment of quality

The Supplementary Figure S1A,B gives details about quality assessment of each study. All included studies received moderately high scores from quality scale. One study reported high risk of patient’s selection and one for flow and timing. Two studies gave high risk scores in index test, and one for reference standard. The studies with high risk bias were less than 10%; the possible bias was from the index test and flow and timing. The unclear risks were less than 30% in Risk of Bias and were almost 50% in Applicability Concerns. The rest of studies give moderate quality scores.

### Pooled results

**Figure 2:**
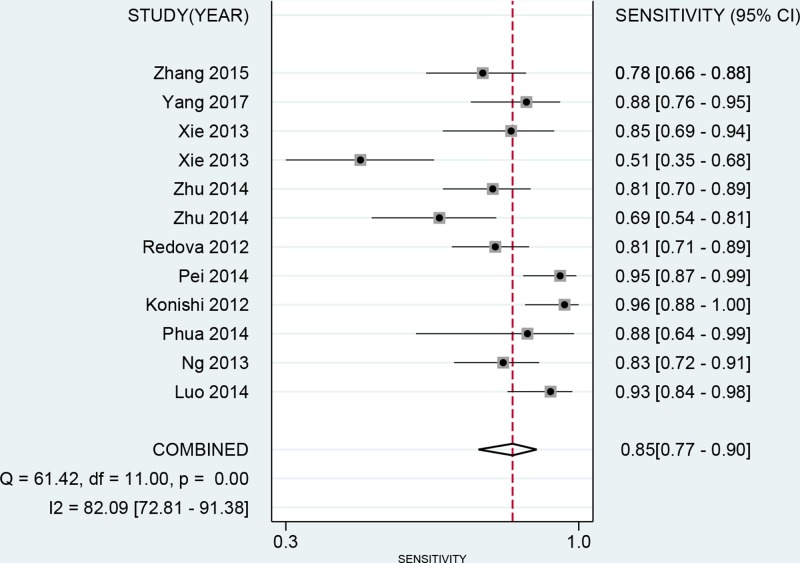
Forest plot of pooled and each study’s sensitivity of miRNA-451 for cancer

**Figure 3:**
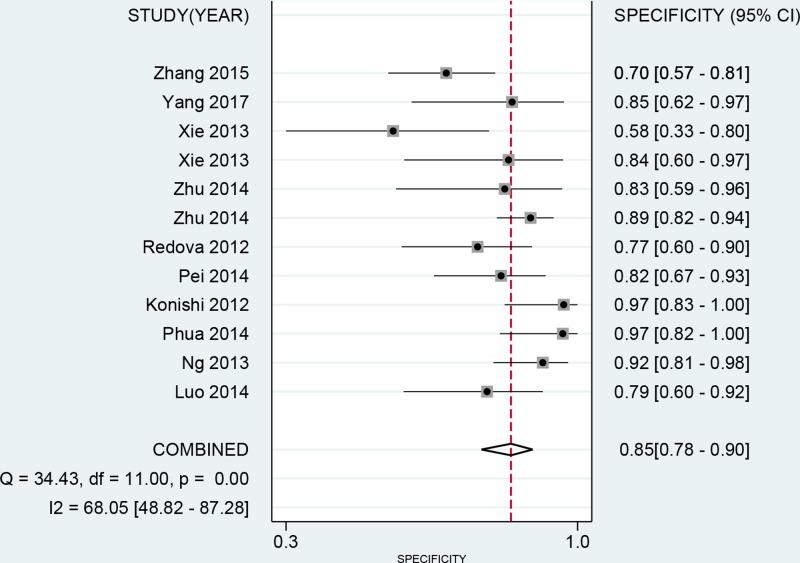
Forest plot of pooled and each study’s specificity of miRNA-451 for cancer

**Figure 4:**
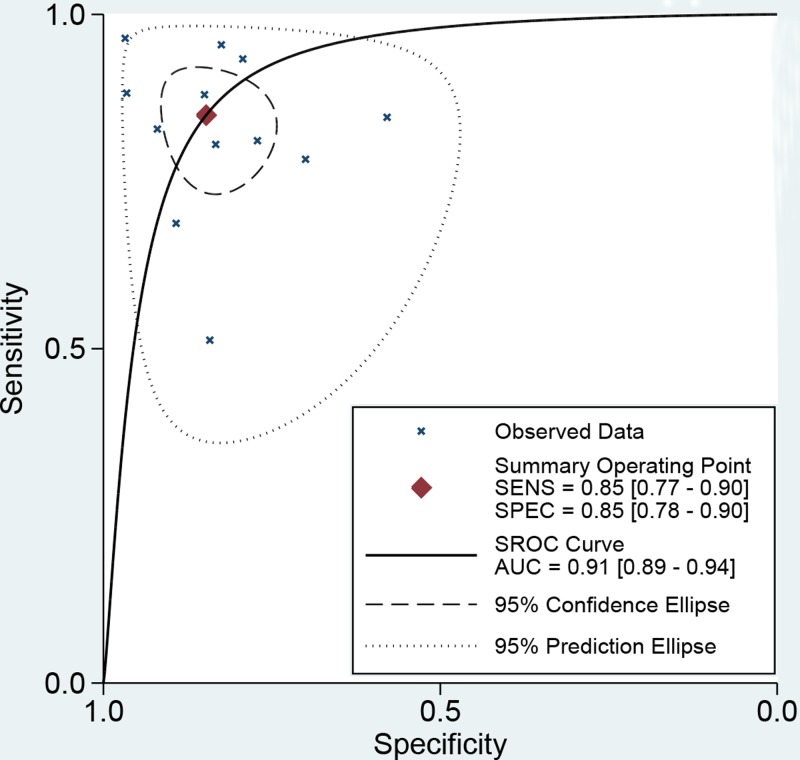
The symmetric receiver operating characteristic curve of miRNA-451 for cancer

**Figure 5:**
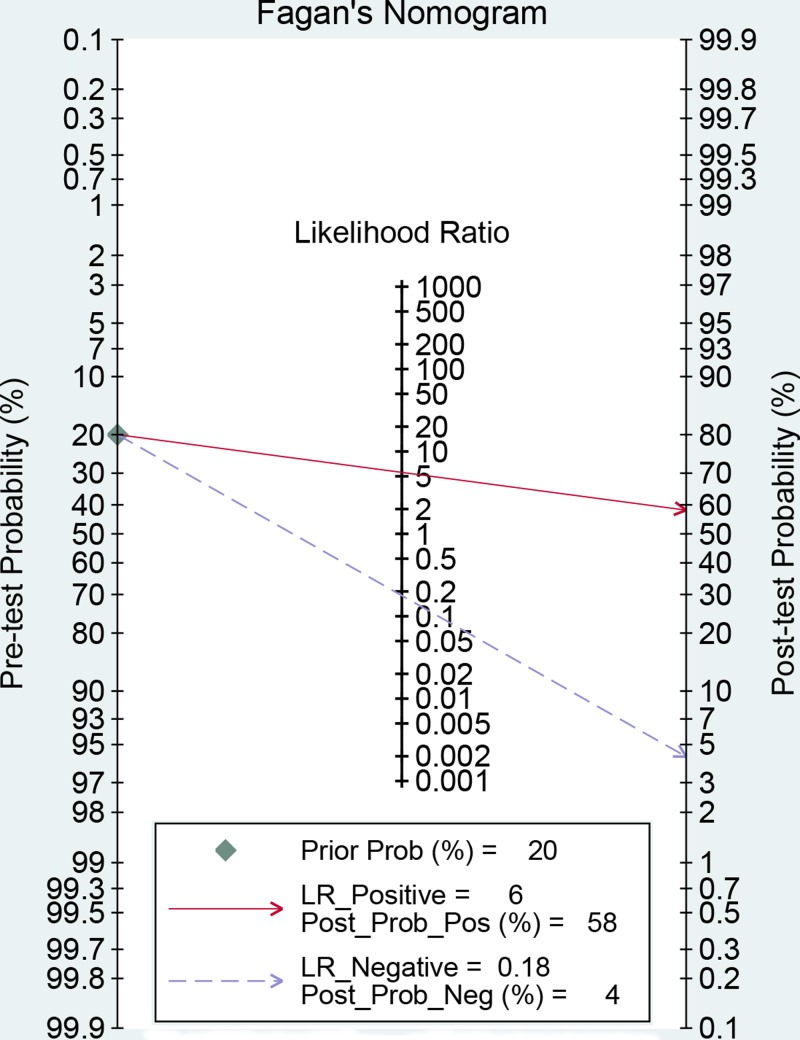
Fagan diagram evaluating the overall diagnostic value of miRNA-451 for cancer (If the pre-test probability is 20% for a patient, the post-test probability will be 59% with a PLR of 6)

No threshold value effect was found for the present studies (*r* = −0.203, *P*=0.527). Besides, as the *I*^2^ value for meta-analysis, we used bivariate random effect model to conduct all analyses. The Table 3 presented the diagnostic ability of miR-451 for human cancers. The whole pooled sensitivity and specificity were 0.85 (0.77–0.90, Figure 2) and 0.85 (0.78–0.90, Figure 3) with their 95% CI, respectively. The pooled area under the cure was 0.91 (95%CI: 0.89–0.94, Figure 4). This index indicates the diagnostic ability of miR-451 was high. We also calculated other parameters as follows: the pooled PLR was 5.57 (95%CI: 3.74–8.31), the NLR was 0.18 (95%CI: 0.11–0.28), and the DOR was 31.33 (95%CI: 15.19–64.61). The Figure 5 gives the prediction of pre-test probability and post-test probability. If the pre-test probability is 20% for a patient, the post-test probability will be 58% with a PLR of 6 and 4% with a NLR of 0.18. All parameters indicated the miR-451 had a high diagnostic ability in detecting cancers.

**Table 3:** Summary estimated of diagnostic performance of miR-451 for cancer detection

Category	SEN (95%CI)	SPE (95%CI)	PLR (95%CI)	NLR (95%CI)	DOR (95%CI)	AUC (95%CI)
**Overall**	0.85 [0.77–0.90]	0.85 [0.78–0.90]	5.57 [3.74–8.31]	0.18 [0.11–0.28]	31.33 [15.19–64.61]	0.91 [0.89–0.94]
**Population**						
Asian	0.85 [0.77–0.91]	0.86 [0.78–0.91]	5.87 [3.78–9.12]	0.17 [0.11–0.28]	34.31 [15.51–75.91]	0.92 [0.89–0.94]
**Cancer type**						
Digestive system	0.83 [0.70–0.91]	0.88 [0.78–0.94]	6.87 [3.40–13.90]	0.20 [0.10–0.37]	35.13 [10.65–115.93]	0.92 [0.90–0.94]
Other types	0.87 [0.79–0.92]	0.81 [0.73–0.87]	4.55 [3.04–6.80]	0.16 [0.10–0.27]	28.51 [12.66–64.20]	0.90 [0.87–0.92]
**Sample source**						
Serum-based	0.87 [0.83–0.90]	0.77 [0.70–0.83]	3.75 [2.69–5.24]	0.16 [0.09–0.28]	27.03 [10.78–67.75]	0.82 [0.66–0.98]
Plasma-based	0.83 [0.74–0.92]	0.91 [0.88–0.95]	8.48 [4.79–15.01]	0.18 [0.11–0.39]	55.18 [17.70–172.07]	0.96 [0.899–1.00]

### Subgroup analyses

We also conducted subgroup analyses among population, cancer type, and sample source to test the stability of results. The pooled sensitivity, specificity, PLR, NLR, DOR, and AUC for subgroup were presented in Table 3. Because nine of ten studies were from Asian, we only give the pooled results among Asian population. The sensitivity and specificity were 0.85 (95%CI: 0.77–0.91) and 0.86 (95%CI: 0.78–0.91), respectively. The PLR and NLR were 5.87 (95%CI: 3.78–9.12) and 0.17 (95%CI: 0.11–0.28), respectively. The DOR and AUC were 34.31 (15.51–75.91) and 0.92 (0.89–0.94), respectively. The pooled sensitivity, specificity, PLR, NLR, DOR, and AUC for digestive system cancer were 0.83, 0.88, 6.87, 0.20, 35.13, and 0.92, respectively. The other cancers were 0.87, 0.81, 4.55, 0.16, 28.51, and 0.90, respectively. For sample source, the results still remained consistent. The corresponding parameters were 0.87, 0.77, 3.75, 0.16, 27.03, and 0.82 and 0.83, 0.91, 8.48, 0.18, 55.18, and 0.97.

### Publication bias

**Figure 6:**
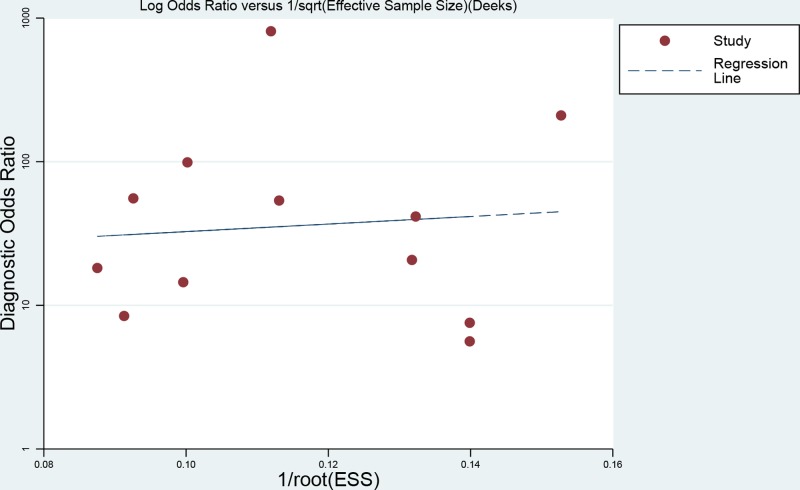
Line regression plot of publication bias (The closer to 0 degree the angel between X-ray gets, the lesser the publication bias gets)

We used the linear regression test of funnel plot asymmetry to assess the publication bias in this meta-analysis. As presented in Figure 6, the *P* value for bias test was 0.781, indicating there was no publication bias for the present meta-analysis.

## Discussion

Our results found that the diagnostic ability of miRNA-451 was moderately high with the pooled sensitivity of 0.85 (95%CI: 0.77–0.90) and specificity of 0.85 (95%CI: 0.78–0.90). The AUC of miRNA-451 was 0.91 (95%CI: 0.89–0.94). This diagnostic accuracy was higher than some biomarkers such as carcinoma-embryonic antigen and squamous-cell carcinoma (with sensitivity of 47.5 and 49 for detecting lung cancer) [22,23]. The miRNA-451 could be a potential tumor biomarker of early-stage cancer detection.

Cancers have been becoming important public health issues around the world. It is urgent to find effective and simple diagnostic methods. In the past several decades, researchers had found many non-invasive methods for cancer detection and screening. MicroRNAs (miRNAs) are short non-coding RNAs that regulate gene expression at the post-transcription level and maintain a dynamic balance of body by regulating cell proliferation, differentiation, and apoptosis. Recently, a lot of studies have revealed that some miRNAs were involved in the tumorigenesis and tumor progression as oncogenes or tumor suppressors [24]. Furthermore, many studies also reported that some miRNAs could be a diagnostic biomarker for cancers [25,26]. The miRNA-451 is located on section of chromosome 17q11.2, which reverses transcription of some proteins with miRNA-144 [27]. Recently, a series of studies reported that there were differences in expression of miRNA-451 in different tumor tissue cells, which indicated the potential diagnostic ability in tumor. Xie found the expression of miRNA-451 was up-regulated in saliva, with the sensitivity of 84.6% and specificity of 57.9% in diagnosing esophagus cancer [18]. Ng also found the expression of serum miRNA-451 was up-regulated in breast cancer patients. The combined positive and negative rates were 88% and 92%, respectively, and the positive rate was 96% in diagnosing early-stage ductal carcinoma *in situ* [9]. Besides, Redova found down-regulated miRNA-451 was observed in the renal carcinoma patients. A combination of miR-378 and miR-451 enables identification of renal cell carcinoma (RCC) serum with the sensitivity of 81% and specificity of 83% [7]. These results indicated that miRNA-451 could be a potential diagnostic marker in tumor. We conducted a comprehensive analysis due to study limitation of individual study. Our results reported higher sensitivity and specificity (85% vs 85%). We also calculated other parameters. The DOR reflects the combination of sensitivity and specificity, ranging from 0 to infinity. The higher the value, the better the diagnostic ability. Our results found that the DOR was 31.33 (95%CI: 15.91–64.61), which means the diagnostic accuracy was high. Otherwise, we also calculated the PLR and NLR. Two indices can assess the clinical application value. The combined PLR of 5.57 means that cancer patients have higher chance by 5.57-fold than those without cancer when miRNA-451 was positive in examination. The combined PLR was 0.18, indicating the probability of patients with cancer was 18% when detection results were negative. However, according to the criteria, only if PLR > 10 and NLR < 0.1, the diagnostic ability was the best and had better ability of confirmation and exclusion [28]. Our results did not conform to the requirements. Previous studies found combination of multi-miRNA can achieve higher diagnostic ability. The higher clinical value could be achieved with combination applications [29,30]. We also conducted subgroup analyses in different populations, cancer types, and sample sources. Similar results were found, indicating the current results were stable and reliable.

Our study still has several limitations. First, we have tried our best to perform systematical searches. Some data may be still ignored such as unpublished study. Second, the included studies were from different cancer types and population settings, which may have some impacts on pooled results because miRNA-451 was low expression in some cancers, and high expression in other cancers. The present results still remained stable in spite of these differences. Third, miRNA-451 had higher diagnostic ability. However, the single miRNA still has some limitations. Combination of multi-miRNA could achieve better utility [31]. Finally, for clinical purpose, it requires more studies and analyses to investigate the mechanism of miRNA-451 in specific cancers, which promoted the application of miRNA-451 in cancer treatment.

In conclusion, our results found that the miRNA-451 has a moderately high diagnostic ability for cancers, and could be a potential early screening biomarker, and considered as an adjuvant diagnostic index when being combined with others clinical examinations. The diagnostic utility did not depend on population, cancer types, and sample sources. The miRNA-451 was of importance in digestive system cancers. Further studies are needed for illustrating mechanism.

## Supporting information

**Supplementary Figure:** 

## Abbreviation

AUC: area under cure
CI: confidence interval
CNKI: China National Knowledge Infrastructure
DOR: diagnostic odds ratio
FN: false negative
FP: false positive
MeSH: Medical Subject Headings
NLR: negative likelihood ratio
PLR: positive likelihood ratio
PRISM: Preferred Reporting Items for Systematic Reviews and Meta-Analyses
ROC: Receiver Operating Characteristic
SROC: summary receiver operator characteristic
TN: true negative
TP: true positive
qRT-PCR: quantitative real time polymerase chain reaction
RCC: renal cell carcinoma
