# Supplementary figures and images for: Clinical utility of microRNA-451 as diagnostic biomarker for human cancers
Source: Biosci Rep. 2019 Jan 15;39(1):BSR20180653. doi: 10.1042/BSR20180653 (PMC6331668; doi:10.1042/BSR20180653)

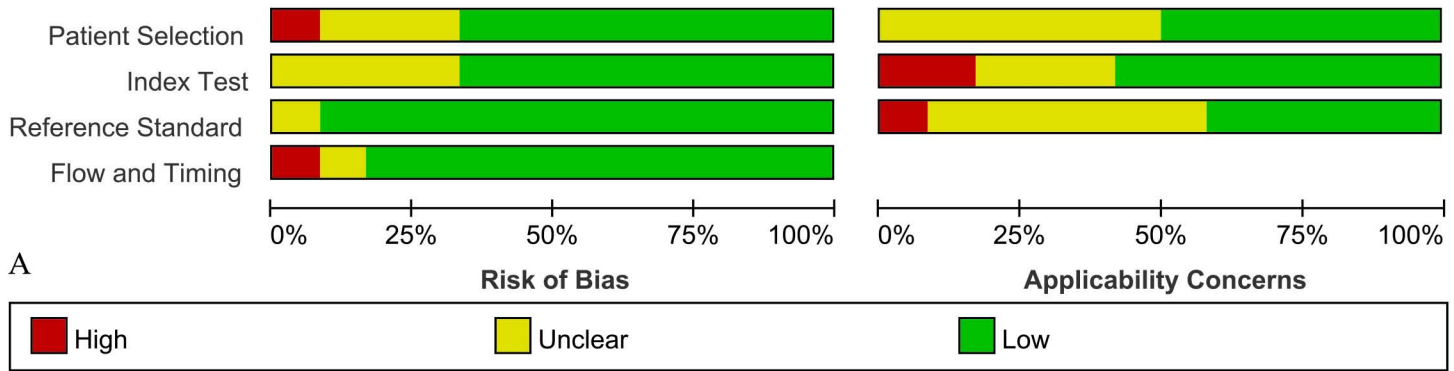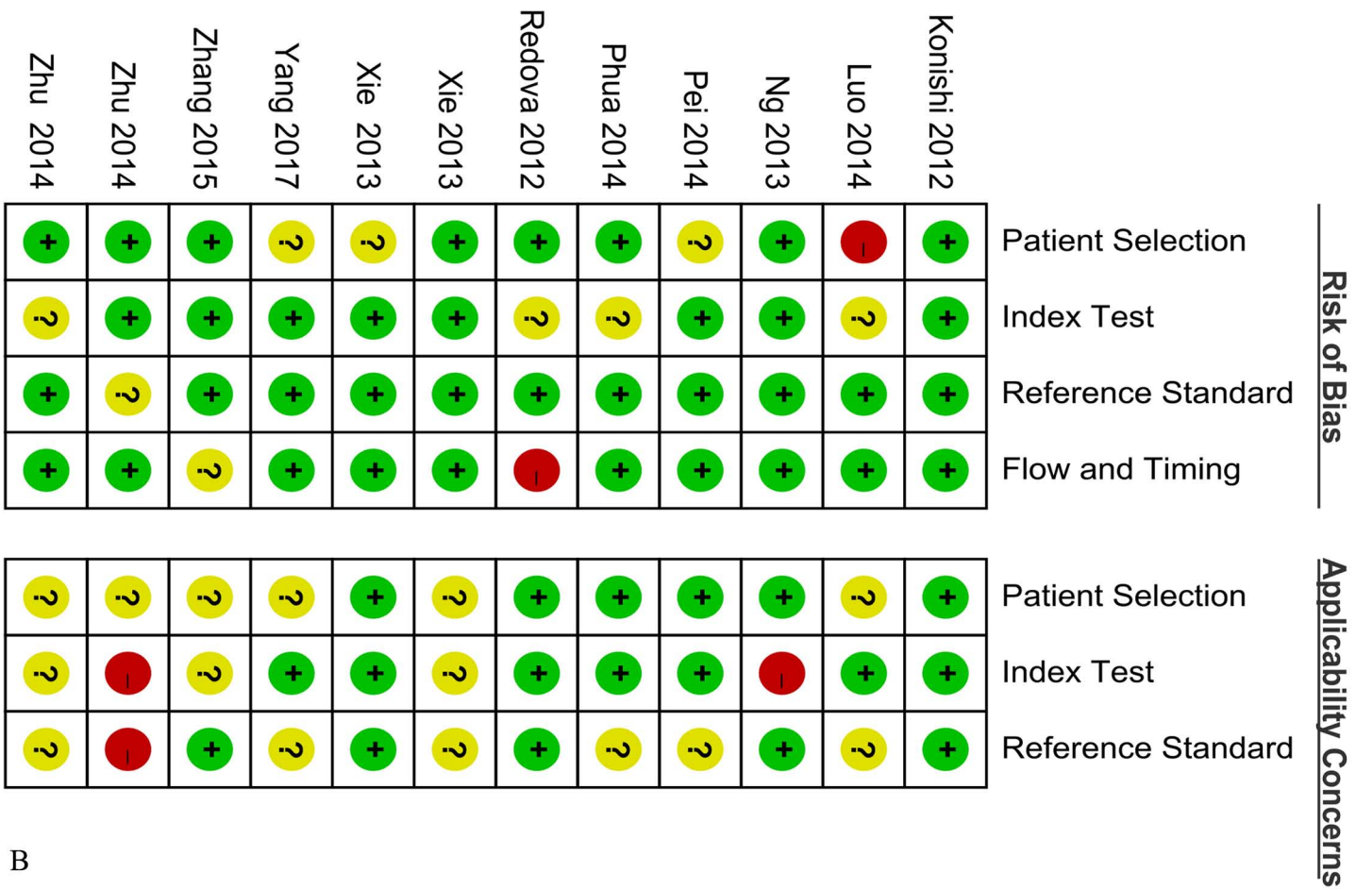

Supplement: Supplementary file 1 [file bsr20180653_Supp1.pdf]
